# PReS-FINAL-2053: Vitamin D receptor polymorphisms in a cohort of Italian patients with juvenile idiopathic arthritis

**DOI:** 10.1186/1546-0096-11-S2-P66

**Published:** 2013-12-05

**Authors:** F Falcini, F Marini, D Rigante, F Bertini, G Lepri, S Stagi, M Matucci Cerinic, ML Brandi

**Affiliations:** 1 Internal Medicine, Rheumatology Section, Transition Clinic, Italy; 2 Surgery and Translational Medicine, University of Florence, Florence, Italy; 3 Institute of Paediatrics, Università Cattolica Sacro Cuore, Rome, Italy; 4 Endocrinology Unit, Anna Meyer Children's Hospital, Florence, Italy; 5 Internal Medicine, Rheumatology Section, University of Florence, Florence, Italy

## Introduction

A role for vitamin D has been hypothesized in generating disease activity for patients with juvenile idiopathic arthritis (JIA): specific polymorphisms of vitamin D receptor (*VDR*) gene have recently been associated with different biologic response to vitamin D itself.

## Objectives

To evaluate *VDR *polymorphisms in patients with JIA in comparison with unrelated healthy controls.

## Methods

We recruited 63 Italian children, adolescents and young adults with JIA (mean age 16.21 + 7.11 SD yrs, 51 female and 12 males, female/male ratio 4.25 from 1 Unit of Paediatric Rheumatology and 1 Unit of Rheumatology, Transition Clinic. After informed consent, during routine laboratory tests, their genomic DNA was extracted from peripheral blood leukocytes, to analyze *VDR *polymorphisms by PCR-based sequencing (*CDX2 *in the promoter region) and PCR-based enzymatic digestions (*FokI *in exon 2, *BsmI *and *ApaI *in intron 8, and *TaqI *in exon 9). An Italian population of 2221 unrelated individuals without JIA was used as healthy controls.

## Results

The distribution of *FokI, BsmI, ApaI*, and *TaqI *polymorphisms did not show significant differences between children with JIA and controls. Regarding the *CDX2 *polymorphism, we observed a statistical difference in the distribution of GG and GA genotypes, with the GG genotype more frequent in JIA subjects (Yates-corrected chi-square 6.97; Odds ratio = 2.08; *p *= 0.008) and the GA genotype in healthy controls (Yates-corrected chi-square 4.04; Odds ratio = 0.55; *p *= 0.044). Data about AA genotype were not significant due to their very low number (three) within the JIA population. G allele resulted to be more frequent in JIA subjects (Yates-corrected chi-square 6.51; Odds ratio = 1.82; *p *= 0.011).

## Conclusion

Pathogenetic mechanisms influencing the predisposition to JIA are poorly elucidated. Our analysis of *CDX2 *polymorphisms located in the promoter region of the *VDR *gene has revealed that both GG genotype and G allele are more represented in patients with JIA. By our preliminary data, we can speculate that the G allele decreases *VDR *transcriptional activity with respect to the A allele, as well as the presence of GG genotype could explain a reduction of *VDR *activity, with subsequent decreased response to vitamin D and potential immunity deregulation leading to JIA.

## Disclosure of interest

None declared.

